# Differences in Synovial Cytokine Profile Associated with Long-Term Clinical Outcomes in Patients with Knee Osteoarthritis Undergoing Corrective Osteotomy with Platelet-Rich Plasma or Stromal Vascular Fraction Post-Treatments

**DOI:** 10.3390/ijms232112835

**Published:** 2022-10-25

**Authors:** Aleksey Prizov, Elena Tchetina, Ilya Eremin, Nikolay Zagorodniy, Andrey Pulin, Evgeniy Belyak, Evgeniy Goncharov, Konstantin Kotenko, Ivan Smyshlyaev, Svetlana Glukhova, Aleksandr Lila

**Affiliations:** 1 Orthopaedic Chair, Peoples Friendship University of Russia, Miklukho-Maklaya Str. 6, 117198 Moscow, Russia; 2 Immunology and Molecular Biology Laboratory, Nasonova Research Institute of Rheumatology, Kashirskoe Shosse 34A, 115522 Moscow, Russia; 3 Petrovsky National Research Center of Surgery, Abrikosovsky lane 2, 11999 Moscow, Russia; 4 National Medical Research Center of Traumatology and Orthopedics n.a. N.N. Priorov, Priorova Str. 10, 127299 Moscow, Russia; 5 Pirogov National Medical and Surgical Center, Nizhnyaya Pervomayskaya Str. 70, 105203 Moscow, Russia; 6 FSBI, Central Clinical Hospital with Outpatient Health Center, Marshala Timoshenko Str. 15, 121359 Moscow, Russia

**Keywords:** knee osteoarthritis, corrective osteotomy, platelet-rich plasma, stromal vascular fraction, synovial fluid, cytokines

## Abstract

Functional outcomes and synovial fluid (SF) cytokine concentrations in response to platelet-rich plasma (PRP) or stromal vascular fraction (SVF) post-treatments following open wedge high tibial osteotomy (HTO) in 20 patients with knee osteoarthritis (OA) were examined. Six weeks after surgery, the knees of 10 patients were injected with autologous PRP (PRP subgroup), while another 10 patients were injected with autologous SVF (SVF subgroup) and monitored for 1.5 years. Pain assessment (VAS score) and functional activity (KOOS, KSS, Outerbridge, and Koshino scores) were applied. PRP subgroup performed better compared with the SVF subgroup according to KOOS, KSS, and VAS scores, while the SVF subgroup demonstrated better results according to Outerbridge and Koshino testing and produced more pronounced cartilage regeneration in the medial condyle and slowed down cartilage destruction in its lateral counterpart. SF was collected before and one week after PRP or SVF injections and tested for concentrations of 41 cytokines (Multiplex Assay). In the PRP subgroup, a significant decrease in IL-6 and CXCL10 synovial concentrations was accompanied by an increase in IL-15, sCD40L, and PDGF-AB/BB amounts. The SVF subgroup demonstrated a significant decrease in synovial TNFα, FLT-3L, MIP-1β, RANTES, and VEGF concentrations while SF concentrations of MCP-1 and FGF2 increased. Both post-treatments have a potential for increased tissue regeneration, presumably due to the downregulation of inflammation and augmentation of synovial growth factor concentrations.

## 1. Introduction

Knee osteoarthritis (KOA) is the most common musculoskeletal disease that primarily affects medial, lateral, and patellofemoral compartments of the joint and results in articular cartilage damage, bony osteophytes formation, and sclerosis of the subchondral bone, which are accompanied by low-grade synovial inflammation and pain [1]. 

KOA treatment involves conservative approaches aiming at the relief of clinical symptoms, while it often ends up in advanced knee OA and further requires total joint replacement [2]. However, to delay or escape a necessity for total knee arthroplasty, corrective medial opening wedge high tibial osteotomy (HTO) using rigid plate fixation with the artificial bone substitute is implicated [3]. This treatment relieves knee pain and slows down the progression of OA due to partial unloading of the medial compartment with correction of mechanical axis that results in joint protection and tissue remodeling [4]. However, treatment outcomes have been shown to gradually deteriorate with time [5]. At the same time, it was also noted that the results of HTO might also be associated with changes in joint biological functioning involving the patient’s capacity for tissue regeneration [6]. Presently, it is well documented that human stem cells, such as adipose mesenchymal stem cells as a part of stromal vascular fraction (SVF) and platelet-rich-plasma (PRP) preparations were capable of repairing and regenerating injured tissues primarily in diseases associated with tissue loss or damage [7,8]. Autologous adipose-derived SVF fraction is a heterogeneous combination of endothelial cells, smooth muscle cells, pre-adipocytes, and adipose-derived stem cells [9]. PRP represents concentrated autologous human platelets in a small amount of plasma and contains major growth factors: platelet-derived growth factor (PDGF), basic fibroblast growth factor (FGF-2), vascular endothelial growth factor (VEGF), insulin growth factor (IGF)-1, and transforming growth factor (TGF) [9]. Intraarticular injection of orthobiologics such as PRP and mesenchymal stem cells (MSCs) in patients undergoing HTO has been shown to be safe and produced good clinical outcomes [10]. These MSCs were obtained either from autologous adipose tissue and injected into the patient’s knee as a part of SVF [11,12] or from the bone marrow, purified, enriched in culture up to 87–95% according to MSCs phenotypic markers followed by the injection into the knee [13]. However, molecular mechanisms of cartilage regeneration in response to PRP or SVF injections associated with HTO in KOA patients have not yet been explored in detail.

Articular cartilage regeneration in KOA is associated with the inhibition of catabolic pro-inflammatory cytokines, primarily members of the interleukins (IL)-1, -17 and tumor necrosis factor (TNF) families and activation of anabolic program that is induced by growth factors such as FGF2, TGFβ, IGF, members of BMP family, and WNT signaling [14,15]. The local inflammation, synovitis, observed in KOA that is evidenced by joint pain, swelling, stiffness, and knee articular cartilage loss [16], is associated with the production of various pro-inflammatory cytokines and chemokines in the synovial fluid (SF). Increased concentrations of these pro-inflammatory mediators are capable of suppressing the activity of synovial anabolic factors [17]. However, a decrease in SF amounts of several biochemical markers: cytokines IL-6 and -8, matrix metalloproteinases (MMPs)- 2, -3, and -13, a cartilage oligomeric matrix protein (COMP), and vascular endothelial growth factor (VEGF) after HTO surgery was demonstrated in a recent study [18]. On the other hand, no comprehensive investigation on the effect of PRP or SVF injections after the HTO surgery on the release of pro-inflammatory mediators in the synovial fluid of patients with knee OA has been conducted so far. 

In the present study, we investigated for the first time the effect of PRP or SVF injections after the HTO surgery on cytokine/growth factor release in the synovial fluid of patients with knee OA and demonstrated differential changes in concentrations of the examined mediators, indicating an involvement of variable mechanisms in articular cartilage regeneration in response to the examined treatments.

## 2. Results

### 2.1. Clinical Parameters in the Examined Subgroups of Subjects with OA before Surgery and after PRP or SVF Treatments 

#### 2.1.1. Clinical Parameters in the Examined Subjects with OA before Surgery

Analysis of the demographic and clinical characteristics of 20 patients prior to HTO surgery revealed that the average age of these patients was 54.5 years (range 39–65 years). The majority of patients demonstrated an increased Body Mass Index (BMI) average of 31.5 (range 19.8–37.9). Only 2 out of 20 examined subjects demonstrated BMI values below 25 kg/m^2^. The KOOS scoring was estimated as 38 (range 19–81), pain according to the VAS index was equal to 7 (range 5–10), and average ROM was 120 (range 95–130) at baseline.

#### 2.1.2. Clinical Parameters in the Examined Subjects with OA after PRP or SVF Treatments

**Figure 1:**
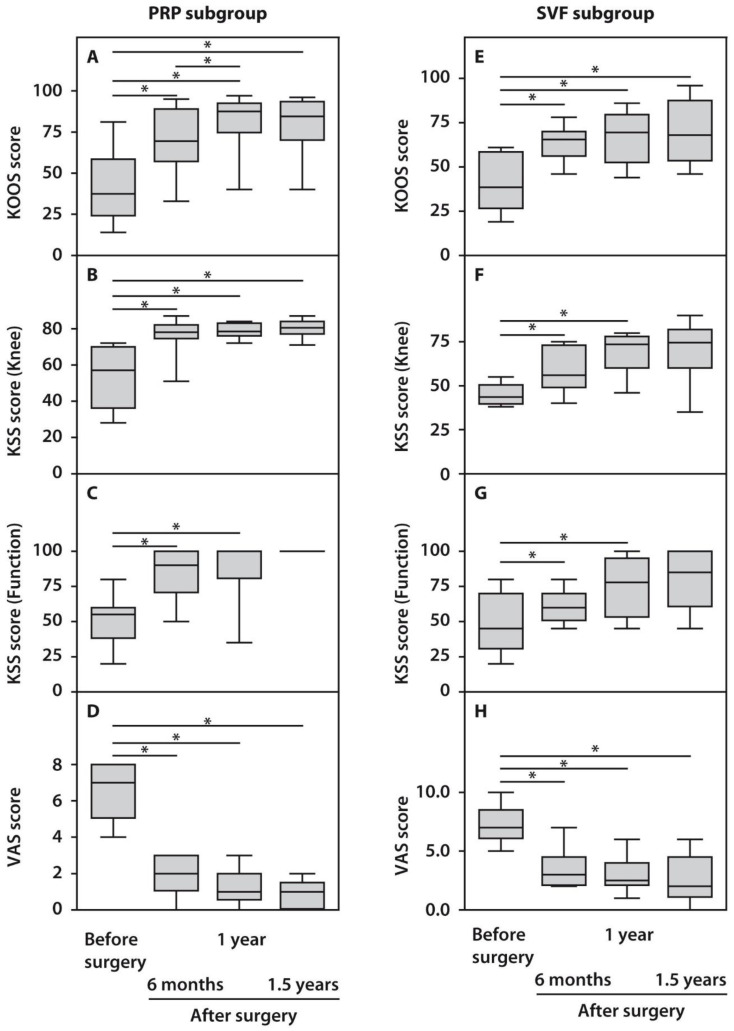
Functional characteristics of patients with OA subjected to HTO therapy with post-surgery injections of PRP (**A**–**D**) or SVF (**E**–**H**) prior to and in the course of follow-up. All statistically significant differences are indicated with an asterisk (*).

The assessment of the obtained clinical indices in response to HTO surgery using the KOOS, KSS, and VAS scores demonstrated statistically significant (*p* < 0.05) functional improvements in the operated knee joints compared to their preoperative status beginning from six months and up to 1.5 years post-surgery (Figure 1). KOOS scores did not demonstrate significant differences between the examined subgroups before surgery, while both subgroups showed significant improvements in the course of follow-up. However, patients from the PRP subgroup performed better one year after surgery compared with the SVF subgroup (Figure 1). Analysis of the KSS scores after surgery demonstrated a less pronounced outcome. However, it was yet significant compared with the preoperative state in both subgroups. KSS recording system also demonstrated significantly higher scores in the case of PRP-treated subjects. Moreover, the recovery of knee joint status and function occurred at a slower pace up to 1.5 years after surgery in the SVF subgroup compared with the PRP subgroup (Figure 1). We also noted significant changes while assessing pain score, which decreased after 1.5 years in both subgroups to minimal values, which no further affected patients’ quality of life (Figure 1). 

#### 2.1.3. Articular Cartilage Status in the Examined Subjects with OA after PRP or SVF Treatments 

Grading of articular cartilage lesions according to Outerbridge classification demonstrated no significant differences prior to surgery in both patient subgroups and in every tested location. However, we observed a significantly higher number of Grade 1 lesions at the lateral femur in patients treated with PRP and significantly more Grade 0 scores in patients treated with SVF at the end of follow-up (Table 1). In addition, at the end of follow-up, partial articular cartilage regeneration (Koshino B-score) was significantly higher in SVF-treated patients compared with that in the PRP subgroup. These results might indicate a higher regeneration potential of intra-articular administration of SVF both in the affected and collateral regions of the joint. Less pronounced regeneration capacity and OA progression at tibial condyles compared to femoral counterparts might be due to a minor contact area associated with their greater loading. Similar results of cartilage regeneration after HTO in combination with PRP or SVF were reported previously [16]; however, the assessment of the lateral joint status during second-look arthroscopy was conducted in our study for the first time. There were no significant differences in the preoperative and postoperative femorotibial angles (MPTA and LDFA) and varus–valgus scores between subgroups.

### 2.2. Synovial Cytokine Levels in the Examined Subgroups before and One Week after PRP or SVF Injection

We did not observe significant differences between synovial concentrations in the majority of the examined cytokines in the study subgroups at baseline (Supplementary Table S1). However, prior to injection SVF subgroup of patients demonstrated significantly higher synovial concentrations of IL-1β and VEGF, while the PRP subgroup showed significantly higher amounts of IL-4 and MCP-3. Cytokines IL-17A, TNFα, IL-1α, IL-9, -2, -3, -5, -13, TGFα, and GM-CSF were not found in the synovial fluids of patients from both subgroups and at both time points supporting previous observations [19]. 

**Figure 2:**
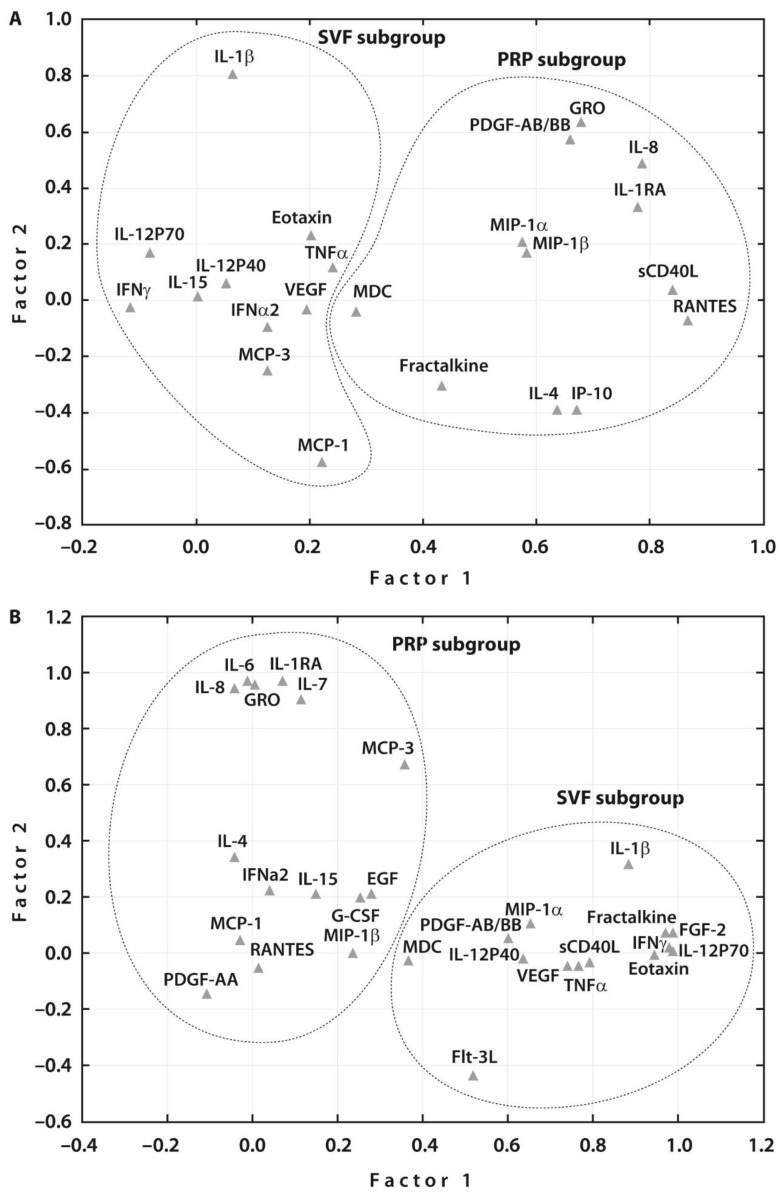
Subgroups of investigated cytokines (*n* = 31) after principal component analysis. (**A**) The factor analysis suggested two main factors, one containing 11 (SVF subgroup) and another 12 (PRP subgroup) cytokines analyzed before treatment; and (**B**) two main factors, one containing 14 (SVF subgroup) and another 15 (PRP subgroup) cytokines analyzed one week after treatment.

Spearman rank correlation analyses related to all the examined cytokines in each treatment group before SVF or PRP injections revealed a significant correlation between the majority of cytokines in both subgroups (Supplementary Tables S2 and S3). These assessments were followed by Multivariate Factor Analysis, which revealed two main groups of the examined cytokines related to each treatment procedure prior to therapy (Figure 2A). One week after therapy, two main groups of the examined cytokines related to each treatment were also noted (Figure 2B). At the same time, the number of cytokines that were associated with PRP or SVF therapies increased after both treatments. Interestingly, both post-treatments resulted in an increased number of the related growth factors, namely, EGF and G-CSF in addition to PDGF-A in case of PRP injection and PDGF-A and FGF2 in addition to VEGF in case of SVF post-treatment. These changes justify the articular cartilage regeneration potential of both post-treatments. 

**Figure 3:**
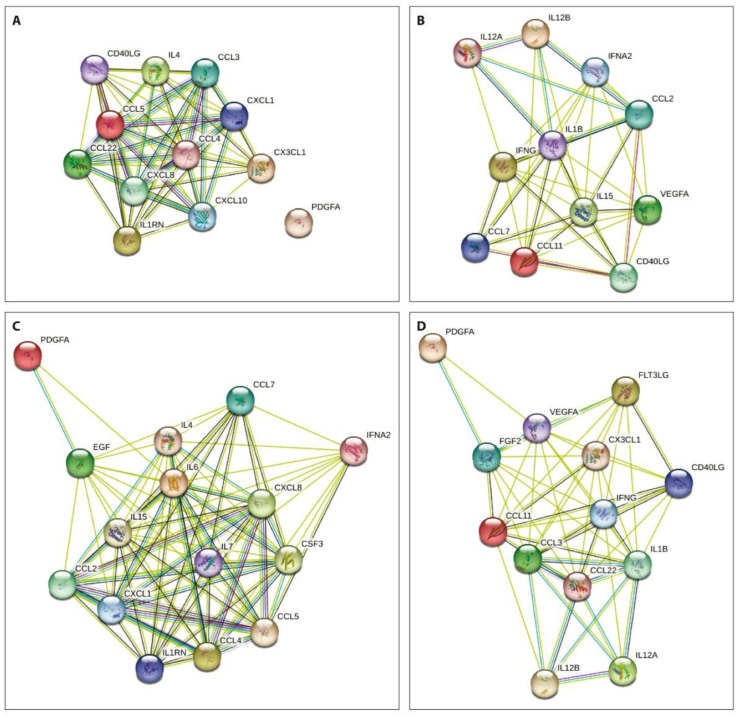
Protein–protein interactions of the examined cytokines prior to (**A**,**B**) and one week after (**C**,**D**) PRP (**A**,**C**) or SVF (**B**,**D**) injections. Nodes show cytokines and the lines previously identified interactions between indicated proteins.

The network analysis of the obtained data using the STRING database aiming to integrate recognized associations between proteins demonstrated that the degree of connectivity was stronger in both patient subgroups before treatment compared to that one week after PRP or SVF injections (Figure 3). 

**Figure 4:**
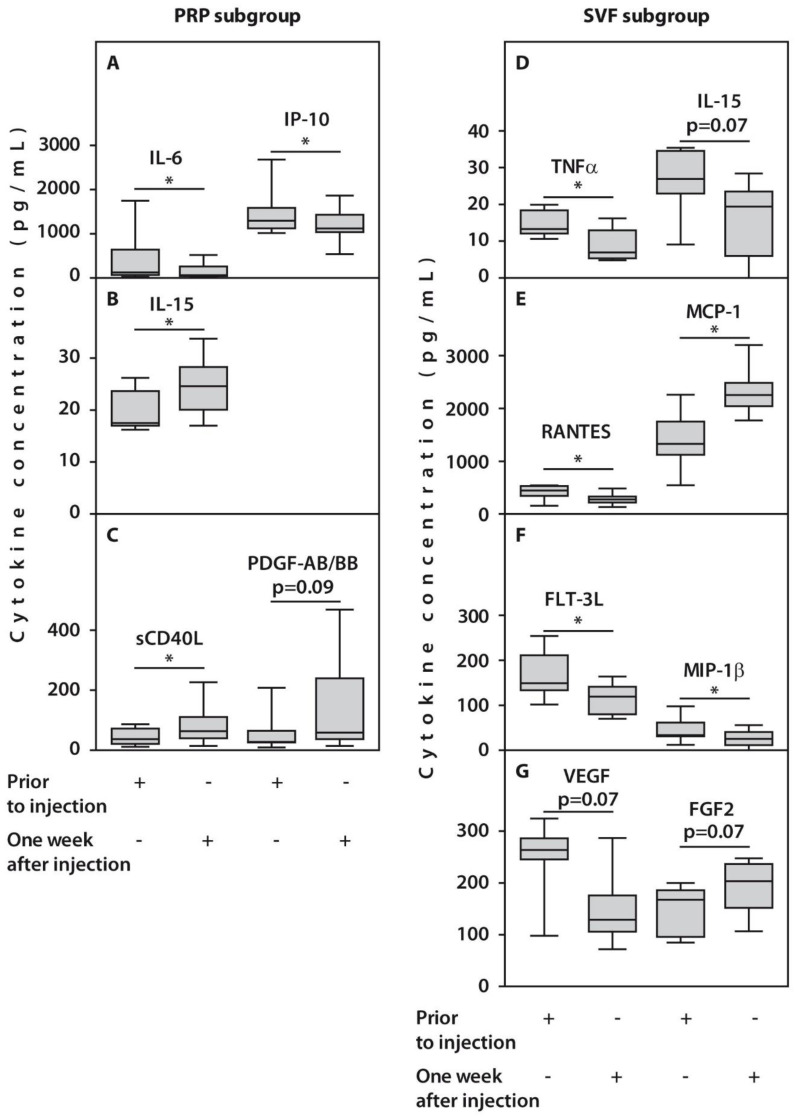
Synovial cytokine concentrations in patients with OA subjected to HTO therapy with post-surgery injections of PRP (**A**–**C**) or SVF (**D**–**G**) prior to the injection or one week after injection. All statistically significant differences are indicated with an asterisk (*). Abbreviations: IL-6, interleukin 6; IP-10, interferon-gamma inducible protein-10; IL-15, interleukin 15; sCD40L, Soluble CD40 ligand; TNFα, tumor necrosis factor alpha; RANTES, Regulated upon Activation, Normal T Cell Expressed and Presumably Secreted; MCP-1, monocyte chemoattractant protein-1; FLT-3L, FMS-like tyrosine kinase 3 ligand; VEGF, vascular endothelial growth factor; FGF2, fibroblast growth factor 2.

In the course of follow-up, no significant changes in the concentrations of 26 and 22 cytokines after one-week post-injection were expressed in patients from PRP and SVF subgroups, respectively. However, we observed alterations in concentrations of several examined cytokines in response to treatment. For example, a significant decrease in IL-6 and IP10 concentrations after one week was noted in synovial fluids from patients treated with PRP (Figure 4, Table 2). This was accompanied by an increase in concentrations of IL-15 and sCD40L (*p* < 0.05) and PDGF-AB/BB (*p* = 0.09). In contrast, we observed a significant (*p* < 0.05) decrease in TNFα, FLT-3L, MIP-1β, RANTES, and VEGF, and an increase in MCP-1 synovial concentrations, while FGF2 amounts demonstrated only a trend for an increase (*p* = 0.07) in patients treated with SVF (Figure 4, Table 2). 

## 3. Discussion

Synovial fluid is secreted by synovial cells and is primarily involved in chondrocyte metabolism and extracellular matrix turnover. Changes in SF cytokine concentrations in the course of OA treatment might be useful indicators of the therapy efficacy as their amounts were significantly correlated with radiographic OA severity and knee pain [20]. Here in our preliminary study, we demonstrated that HTO surgery combined with PRP or SVF post-injections resulted in significant improvements in functional results and pain score and support recent observations on injections of either PRP or MSCs from autologous adipose tissue into the joints of patients with minimal or medial (KL I-III) knee OA [21]. Moreover, we observed that the overall clinical outcome was significantly more pronounced in the case of PRP post-treatment compared to SVF injection throughout the follow-up period. At the same time, a combination of SVF and PRP has been shown to be even more efficient compared with PRP alone after HTO therapy [16]. It is also worth noting that the functional outcomes after 2 years following HTO obtained in our study are in agreement with that demonstrated by others [22,23].

Pain sensation and clinical manifestations of the OA disease were previously associated with an increase in cytokine expression [24,25]. Therefore, a significant decrease in SF concentrations of IL-6 and IP-10 in case of PRP treatment as well as TNFα, IL-15, Flt-3L, MIP-1β, RANTES, and VEGF in case of SVF injection in association with reduction of pain scores that were observed in our study further support this finding. Indeed, synovial fluid IL-6 concentrations positively correlated with pain in post-traumatic OA [26], while a significant correlation between a decrease in serum IP-10 levels and pain reduction was also previously reported [27]. It is worth noting that after PRP treatment of the examined patients, synovial IL-6 amounts decreased to concentrations observed in healthy subjects [28]. This observation is important as IL-6 signaling is required for chondrocyte proliferation and anabolism [29]. 

Autologous adipose tissue multipotent mesenchymal stromal cells have previously been shown as an efficient biological treatment [30]. However, SVF preparations that undergo minimal manipulation during isolation and contain heterogeneous cell populations could have a higher potential for tissue regeneration compared with isolated stem cells and, therefore, may be more efficient in a clinical setting [31]. Indeed, improvements in the clinical scores in response to SVF injection followed by HTO surgery were associated with cytokine downregulation in SF of the examined patients with OA. Among these was TNFα, which is capable of activating endothelial cells and recruiting pro-inflammatory cytokines such as IL-6 and IL-1β and promoting osteoclast differentiation associated with the OA severity, joint space narrowing, and cartilage loss [32].

SVF cells injected into patient knees after HTO surgery in this study produced changes in amounts of several chemokine ligands (CCL), which are, together with their receptors, capable of modulating monocyte/macrophage recruitment in multiple inflammatory diseases [33]. For example, positive clinical outcomes might be associated with the observed decrease in amounts of MIP-1β and RANTES in response to SVF injection as increased concentrations of these chemokines were linked to increased type II collagen degradation due to activation of MMP-1 and -13 expressions in SF [34]. 

In contrast, an increase in MCP-1 (CCL2) concentration in the synovial fluid of the examined patients with OA followed by SVF injection might account for higher pain scores compared with PRP treated subgroup as previously MCP-1 concentrations in SF were positively associated with pain in patients with OA and in animal studies [35] pointing to the central role of CCL2/CCR2 axis in the development of pain hypersensitivity [36]. On the other hand, MCP-1 upregulation is critically involved in monocyte mobilization and might be required for stromal cell activation in SVF preparations [37]. 

Downregulation of the knee joint inflammation and pain indices in response to HTO in the case of both post-treatments was associated with partial regeneration of articular cartilage in the medial part of the joint and slowing down its destruction in its lateral part. These changes might involve increased growth factor activity [38]. For example, reparative changes in the knee joints in the examined patients after HTO surgery combined with PRP might also result from an increase in amounts of PDGF-AB/BB, which is a systemic growth factor released during platelet aggregation and is capable of stimulating bone remodeling, extracellular matrix production, and revascularization [39]. Another cytokine upregulated in the SF in response to PRP injection was sCD40L, which is a potent immunomodulator [40]. sCD40L might also be involved in regeneration processes as PDGF-AB/BB, and sCD40L belongs to a single cluster of interrelated SF mediators associated predominantly with growth factors [19]. Increased synovial fluid amounts of IL-15 in response to PRP treatment is an interesting outcome as it was associated with early knee OA [41], indicating the presumable recapitulation of the joint degradation to a healthier phenotype.

The regenerative potential of SVF cells is reflected by a trend for FGF2 upregulation, which is involved in angiogenesis and mesenchymal cell mitogenesis and promotes the regeneration of articular cartilage [38,42]. In addition, recombinant FGF2 has been shown to accelerate bone healing after the closing wedge HTO in human studies [42]. 

Multivariate Factor Analysis demonstrated that two main groups of the examined cytokines were related to each treatment procedure both prior to and one week after therapy. Moreover, the degree of connectivity was stronger in both patient subgroups before treatment compared to that one week after PRP or SVF injections. Similar associations were observed in a recent study on synovial cytokine concentration changes during hyperacute serum treatment in human OA knee joints [43].

## 4. Materials and Methods

### 4.1. Patients

Twenty patients with knee OA (median age 54 years) median disease duration of 61.2 months were examined. All of the examined patients fulfilled the criteria of the American College of Rheumatology regarding OA [44]. The study was conducted according to the guidelines of the Declaration of Helsinki and approved by the local ethical committee (Buyanov V.M. Moscow City Clinical Hospital), Minutes No. 06-07.04.17 from 7 April 2017. All patients signed the written informed consent form.

Inclusion criteria were as follows: unrelated patients with knee OA aged from 20 to 65 years old who visited Buyanov V.M. Moscow City Clinical Hospital between January 2020 and December 2021. These patients had body mass index <40 kg/m^2^, and experienced pain in a knee joint ≥40 mm according to the VAS scale longer than half a day. They had a range of motion (ROM) in a >90°, flexion contracture less than 10°. These patients had radiographic osteoarthritis of the medial compartment with Kellgren–Lawrence OA grades of II-III, the absence or initial osteoarthritis of the lateral joint compartment and patella-femoral joint, and varus deformity >3° and <14°. 

Exclusion criteria: secondary osteoarthritis of the knee joint: post-traumatic (caused by clinically significant and documented trauma), intra-articular fracture with clinically significant post-traumatic deformity of the lower limb, septic arthritis, inflammatory joint diseases, gout, severe chondrocalcinosis, Paget’s disease, ochronosis, acromegaly, hematochromatosis, Wilson’s disease, primary osteochondromatosis, osteonecrosis, hemophilia; chondromalacia of the articular cartilage of the lateral knee joint higher than Grade 2 according to Outerbridge classification, the presence of damage to the lateral meniscus higher than Stage 2 according to Stoller classification [45] the angle of varus deformity of the lower limb more than 14° and less than 3°, chronic concomitant somatic diseases in the decompensation stage, such as diabetes mellitus, hypertension, etc., restriction of extension in the knee joint ≥10°, systemic disease in the anamnesis, indications for the initiation of immunosuppressive therapy, venous thromboembolism in the anamnesis (including pulmonary embolism) or a high risk of venous thromboembolism; significant body weight loss (>10%) of unknown etiology in the previous year; patients treated with drugs with a proven effect on the metabolism of cartilage, bones and adipose tissue; the presence of chronic subcompensated or decompensated diseases; clinically significant deviations in the results of laboratory tests; conditions limiting participation in the study (dementia, neuropsychiatric diseases, drug addiction, alcoholism, etc.), participation in other clinical trials 3 months before the onset of the study; patients with malignant tumors, including the post-operative oncological period, including chemotherapy and/or radiation therapy; an increase in Activated Partial Thromboplastin Time (APTT) >1.8 fold; patients with a history of heterotopic ossification; patients who received glycoprotein IIB/IIIA inhibitors before the study. 

### 4.2. Clinical and Radiographic Testing

Clinical characteristics were assessed prior to surgery, at six months, one year, and 1.5 years after surgery. The pain was evaluated using a visual analog scale (VAS) score. The Knee Injury and Osteoarthritis Outcome Score (KOOS) questionnaire was applied for functional activity assessment [46]. Knee Society Score (KSS) was used for knee joint score (pain, range of motion, and stability) and functional score (walking distance and ability to climb stairs) [47] Outerbridge classification and Koshino scores were used for grading of articular cartilage lesions [48]. Medial proximal tibial angle (MPTA) and lateral distal -femoral angle (LDFA) were measured using standing AP radiographs taken immediately before surgery, on surgical removal of the plate, and after 6 months, one year, and 1.5 years after surgery. 

### 4.3. Surgical Techniques

Open wedge high tibial osteotomy was performed as described previously [49]. In brief, the surgery was conducted under spinal anesthesia. Initially, arthroscopic lavage and debridement of the knee joints were implemented. After an oblique incision in a projection of tibial medial condile 5–6 cm long, the bone was cut up to the lateral cortical region avoiding damage to the cortex. The osteotomy wedge was opened to a certain size at the preoperative period corresponding to the size of the tricalcium phosphate block. After that, an electron-optical converter control was performed. Then a β-tricalcium phosphate wedge (Biosorb, Lourdes, France) was implanted with fixation of the osteotomy zone using a locking plate (Otis + SBM, France). During the post-operative period, no immobilization of the knee joints was used, aiming early activation and recovery of knee motion. No loading on the operated joint was allowed up to six weeks after surgery. Six weeks after surgery, patients were divided into 2 subgroups: the knees of 10 of these patients were injected with autologous PRP preparation (0.9 × 10^6^ cells/μL of plasma) (PRP subgroup) while another 10 subjects with knee OA were injected with autologous SVF preparation (1.6 × 10^8^ cells per knee) (SVF subgroup). 

### 4.4. Platelet-Rich Plasma (PRP) Preparation

Six weeks after surgery, patients from the PRP subgroup underwent blood sampling from the cubital vein in a volume of 40 mL, then 2 mL of PRP was obtained by double centrifugation 130× *g* for 15 min at room temperature (RT) to separate erythrocytes followed by 250× *g* for 15 min at RT to concentrate platelets as described previously [50]. Then, 2 mL of autologous PRP preparation was injected into the patient knee joint.

### 4.5. Stromal Vascular Fraction (SVF) Preparation 

Six weeks after surgery, patients from the SVF subgroup underwent local anesthesia. A team of plastic surgeons performed paraumbilical access to withdraw adipose tissue with a syringe from the anterior abdominal wall in a volume of 150–200 mL. Celution 800/CRS device (Cytori Therapeutics Inc., San Diego, CA, USA) and enzymatic digestion by Cellase (Cytori Therapeutics Inc., San Diego, CA, USA) were applied for extraction of SVF fraction from autologous fat tissue, cell counting, and assessment of their viability in accordance with manufacturer’s recommendations. Then, 3.5 mL of SVF preparation was injected into the knee joints. 

### 4.6. Synovial Fluid (SF) Preparation

Six weeks after HTO surgery, paired SF samples were obtained from the operated knee joint immediately prior to PRP or SVF injection, followed by a second withdrawal of the synovial fluid one week later. The samples were immediately centrifuged for 15 min at 3000× *g* at 4°C, and the supernatant was stored at −80°C until use. 

### 4.7. Cytokine Assay 

MILLIPLEX MAP Human Cytokine/Chemokine Magnetic Bead Panel-Premixed 41 Plex-Immunology Multiplex Assay (Millipore, Jaffrey, NH, USA) was used to analyze the cytokine secretion array in synovial fluid samples according to the manufacturer’s recommendations. Fifty microliters of the synovial sample were used for cytokine analysis. Results were registered using a Magpix device (Luminex platform, Austin, TX, USA) and analyzed with xPonent software (Luminex, Austin, TX, USA). Concentrations (pg/mL) of the following cytokines: sCD40L, EGF, Eotaxin/CCL11, FGF-2, Flt-3 ligand, Fractalkine, G-CSF, GM-CSF, GRO, IFN-α2, IFN-γ, IL-1α, IL-1β, IL-1ra, IL-2, IL-3, IL-4, IL-5, IL-6, IL-7, IL-8, IL-9, IL-10, IL-12 (p40), IL-12 (p70), IL-13, IL-15, IL-17A, IP-10, MCP-1, MCP-3, MDC (CCL22), MIP-1α, MIP-1β, PDGF-AAPDGF-AB/BB, RANTES, TGF-α, TNF-α, TNF-β, VEGF were measured.

### 4.8. Statistical Analysis 

Statistical analyses were performed using the Statistica software package version 12.0 (StatSoft Inc., Tulsa, OK, USA). The Mann–Whitney U test was applied for the analysis of differences between subgroups. Wilcoxon signed-rank test was used for within-subgroup analyses. Quantitative data were expressed as medians [IQR, 25th; 75th percentiles]. *p* value ≤ 0.05 was considered statistically significant. All statistically significant differences are indicated with an asterisk (*).

## 5. Conclusions

In summary, we conducted for the first time a pilot study of synovial cytokine profile analyses associated with the assessment of long-term clinical outcomes in patients with knee osteoarthritis undergoing corrective osteotomy with platelet-rich plasma or stromal vascular fraction post-treatments. These outcomes are mechanistically related to molecular changes that occur in the individual cells and tissues. Hence, we examined alterations in cytokine profiles in the synovial fluid, which is one of the main players in destructive inflammatory activities that resulted in articular cartilage degeneration and knee joint destruction. Our preliminary study also demonstrated that HTO surgery combined with PRP or SVF post-injections resulted in significant improvements in functional outcomes in patients with osteoarthritis. Intra-articular administration of SVF produced more pronounced improvements related to cartilage regeneration in the medial condyle and slowed down the destruction of articular cartilage in its lateral part, being more prominent in the femoral articular surface. However, PRP post-injection resulted in a better functional outcome and pain control compared with SVF injection to the patient knees. These effects were associated with changes in expression of pro-inflammatory cytokine concentrations in the synovial fluid in response to both treatments. Moreover, a potential for increased tissue regeneration capacity of PRP and SVF post-treatments might be due to augmentation of synovial PDGF AB/BB and FGF2 amounts, respectively. At the same time, the molecular mechanisms involved in articular cartilage regeneration might be different in the case of PRP or SVF administration, as differential changes in cytokine/chemokine synovial fluid concentrations were noted. These assessments might reveal the intrinsic mechanisms early in the disease and, therefore, might help develop innovative means for disease treatment. Further research involving larger patient cohorts is required to validate our findings and to conduct a more detailed investigation of the cellular and molecular mechanisms of articular cartilage regeneration in response to HTO surgery which might provide new approaches for better disease control.

## Figures and Tables

**Table 1:** Clinical and functional characteristics of patients with OA subjected to HTO therapy with post-surgery injections of PRP or SVF prior to and in the course of follow-up.

	PRP Subgroup (*n* = 10) Me [IQR]	SVF Subgroup (*n* = 10) Me [IQR]	*P* (Mann–Whitney U-Test)
Age, years	56.5 [52.5; 63.5]	52.5 [45.0; 57.0]	n.s.
BMI, kg/m^2^	30.2 [26.5; 33.2]	32.8 [25.2; 34.9]	n.s.
Outerbridge score			
Medial femur			
before surgery			
4	9	9	-
3	1	1	-
Medial femur			
1.5 years after surgery			
4	7	9	n.s.
3	3	1	n.s.
Lateral femur			
before surgery			
1	3	1	n.s.
0	7	9	n.s.
Lateral femur			
1.5 years after surgery			
2	1	1	-
1	4	0	-
0	5	9	0.05 *
Medial tibia			
before surgery			
4	8	8	-
3	2	2	-
Medial tibia			
1.5 years after surgery			
4	6	6	-
3	3	4	n.s.
2	1	0	-
Lateral tibia			
before surgery			
1	10	8	n.s.
0	0	2	-
Lateral tibia			
1.5 years after surgery 2			
1	3	3	-
0	7	6	n.s.
	0	1	-
Koshino score			
1.5 years after surgery			
Medial femur			
C2	0	0	-
C1	1	1	-
B	2	7	0.02 *
A	6	2	n.s.
Lateral femur			
A	10	10	-
Medial tibia			
C2	2	2	-
C1	1	1	-
B	3	4	n.s.
A	4	3	n.s.
Lateral tibia			
A	10	10	-
MPTA (°)			
Before surgery	84.5 [83.5; 85.5]	85.5 [84.6; 87.3]	n.s.
1.5 years after surgery	91.8 [90.7; 94.0]	90.7 [90.3; 92.9]	n.s.
	*p ≤* 0.01 *	*p ≤* 0.01 *	
Varus–Valgus (°)			
Varus before surgery	8.3 [5; 11]	6.2 [5; 7]	n.s.
Valgus 1.5 years after surgery	1.6 [0.5; 3.5]	1.2 [0.2; 3.35]	n.s.

Note: Me [IQR], median [interquartile range, 25th, 75th percentiles]. All statistically significant differences are indicated with an asterisk (*).

**Table 2:** Changes in synovial fluid cytokine concentrations (pg/mL) in patients with OA subjected to HTO therapy prior to post-surgery injections of PRP or SVF and one week later.

	Cytokine	Median Change	%Change
PRP subgroup	IL-6	59.7	−47.8
	IL-15	7.1	+40.5
	sCD40L	27.2	+74.3
	IP-10	178	−13.8
	PDGF-AB/BB	30.8	+110.7
SVF subgroup	TNFα	6.4	−48.2
	IL-15	7.5	−27.9
	Flt-3L	25.6	−23.9
	MIP-1β	8.4	−24.9
	RANTES	169.9	−38.1
	MCP-1	928	+69.8
	VEGF	137.8	−51.1
	FGF2	35.6	+21.2

## Data Availability

The data presented in this study are available on request from the corresponding author.

## Supplementary Materials

The following supporting information can be downloaded at: https://www.mdpi.com/article/10.3390/ijms232112835/s1.
