# Supplementary material for: Differences in Synovial Cytokine Profile Associated with Long-Term Clinical Outcomes in Patients with Knee Osteoarthritis Undergoing Corrective Osteotomy with Platelet-Rich Plasma or Stromal Vascular Fraction Post-Treatments
Source: Int J Mol Sci. 2022 Oct 25;23(21):12835. doi: 10.3390/ijms232112835 (PMC9657490; doi:10.3390/ijms232112835)
Supplement: Supplementary file 1 [file ijms-23-12835-s001.zip › ijms-1958086-supplementary.pdf]

Table S1. Synovial fluid cytokine concentrations (pg/mL) in the examined patients with OA prior to HTO surgery

**Cytokine**	**SVF subgroup at baseline (n=10) Me [IQR]**	**PRP subgroup at baseline (n=10) Me [IQR]**	***P* (Mann-Whitney U-test)**
IFNγ	6.9 [0.0;13.8]	0.0 [0.0;4.1]	n.s.
IL12P40	13.8 [3.8;32.4]	21.8 [10.7;38.4]	n.s.
IL-12P70	4.5 [0.0;7.1]	3.0 [0.0;4.3]	n.s.
IL-1β	58.5 [42.5;96.0]	0.0 [0.0; 6.8]	<0.01*
IL-6	373.8 [142.7;715.7]	125.1 [45.3;644.9]	n.s.
IL-7	30.4 [14.3;39.5]	9.0 [2.3; 37.6]	n.s.
TNFα	13.3 [11.7;18.4]	12.2 [9.8;16.3]	n.s.
IL-10	12.4 [9.6; 14.4]	8.9 [4.4; 15.6]	n.s.
IL-1RA	36.4 [26.0; 56.0]	40.9 [25.3;56.5]	n.s.
IL-4	6.9 [0.0; 11.7]	58.4 [10.4; 87.0]	<0.01*
EGF	0.0 [0.0; 35.3]	16.4 [0.0;23.8]	n.s
FGF-2	167.5 [93.5;186.0]	171.1 [100.7; 187.7]	n.s
G-CSF	14.6 [9.3;24.5]	19.0 [8.0;28.0]	n.s
Flt-3L	149.2 [131.2;211.6]	149.6 [108.9;167.4]	0.60
PDGF-AA	21.2 [20.3;27.9]	14.4 [8.0;26.0]	n.s
PDGF-AB/BB	39.6 [27.2;61.8]	27.8 [22.2;64.5]	n.s
VEGF	263.9 [243.5;286.0]	103.0 [61.7; 167.7]	<0.01*
Eotaxin	71.9 [52.8;81.9]	54.5 [43.9; 60.5]	n.s
Fractalkine	74.2 [68.9;84.9]	133.2 [87.4;176.9]	0.07
GRO	189.5 [168.7;242.1]	190.1 [137.3;358.1]	1.00
MCP-3	45.8 [33.9;66.5]	93.1 [61.3; 128.1]	0.01*
MDC	658.1 [289.3;942.4]	558.2 [480.0; 782.1]	0.80
IL-8	318.0 [89.2;425.3]	189.3 [76.5;304.1]	n.s
IP-10	1220.0 [950.6;1569.0]	1295.0 [1100.0;2680]	n.s
MCP-1	1328.0 [1100.0;1755.0]	1767 [1074;2357]	n.s
MIP-1α	12.4 [0.0;17.2]	18.2 [10.0;30.0]	n.s

MIP-1β	33.8 [28.8;61.5]	40.9 [29.4;51.6]	0.60
RANTES	445.6 [325.5;532.1]	631.6 [332.4;1770.0]	n.s
IFNα2	16.9 [4.9;47.7]	25.7 [21.0;37.6]	n.s
IL-15	26.9 [22.6;34.5]	17.5 [16.8;23.6]	0.07
sCD40L	21.3 [17.9;27.0]	36.6 [17.9;72.0]	n.s

Note: Me [IQR], median [interquartile range, 25th, 75th percentiles]

Table S2. Spearman's rank correlation coefficients (r) ($p<0.05$) are shown for cytokine concentrations observed in the synovial fluid of patients with OA at baseline prior to SVF injection.

	IFNγ	IL-12P40	IL-12P70	IL-6	TNFα	PDGFAB/BB	Eotaxin	Fractalkine	GRO	MCP-3	IL-8	MIP-1β	IFNα2	IL-15	IP-10
IL-1β	0.85		0.85												
IL-7				0.90											
IL-10				0.83											
Eotaxin		0.73													
Fractalkine	0.82		0.82												
GRO						-0.90									
IL-8		0.83					0.73								
MIP-1α		0.76								0.75	0.78				
IFNα2	0.80		0.80			0.73		0.89				0.73			
IL-15					0.90							0.76			
sCD40L				-0.71	0.74							0.74	0.74	0.74	
IP-10										0.82					
MCP-1				-0.71		0.71			-0.85						
MDC															0.85

Table S3. Spearman's rank correlation coefficients (r) ($p<0.05$) are shown for cytokine concentrations observed in the synovial fluid of patients with OA at baseline prior to PRP injection.

	IFNγ	IL-12P40	IL-12P70	IL-1β	IL-6	IL-7	TNFα	IL-1RA	PDGFAB/BB	VEGF	Eotaxin	Fractalkine	GRO	IL-8	MIP-1α	MIP-1β	RANTES	sCD40L	IP-10
IL-1β	0.80																		
TNFα	0.85			0.78															
IL-10						0.71													
PDGF-AB/BB				0.73	0.66														
Eotaxin				0.67					0.66	0.71									
Fractalkine										0.71									
GRO			0.69	0.76		0.67					0.86								
MCP-3					-0.75														
IL-8					0.96					0.71	0.66								
MIP-1α		0.66	0.77								0.71		0.73						
MIP-1β		0.70						0.83							0.73				
RANTES					0.70					0.73	0.78			0.75					
IFNα2		0.85										0.76				0.66			
IL-15																			
sCD40L								0.71	0.75	0.83									
IP-10										0.76							0.71	0.76	
MCP-1												0.78							0.70
MDC			0.80				0.75				0.88		0.78				0.81		
